# HAART: A Cost-Effective Option for South Africa

**DOI:** 10.1371/journal.pmed.0030037

**Published:** 2005-12-06

There were an estimated 370,000 AIDS deaths in South Africa in 2003 alone. It is, therefore, not surprising that the apparent reluctance of the South African government to support the provision of antiretroviral treatment to people with HIV/AIDS has been the subject of much controversy internationally. The situation is, however, changing, and South Africa is now seeing a scaling up of access to highly active antiretroviral therapy (HAART) and a gradual reduction in HAART prices.

HAART, nevertheless, remains an expensive option, and one that many low-income countries are unable to afford. South Africa is better placed than most sub-Saharan nations to increase access to HAART, but it is clearly essential to establish how much this will cost the country's public health sector and what will be the benefits. It is unfortunate, therefore, that cost-effectiveness studies on HAART have so far been limited to the developed world.

In the January issue of *PLoS Medicine*, Motasim Badri and colleagues publish a study of the use of the cost-effectiveness of HAART conducted in South Africa. During the study period (January 1995 to December 2000), HAART was not available in the publicly funded South African health-care sector.

The research was funded by Secure the Future—a Bristol-Myers Squibb initiative to provide resources for capacity building and for the search for sustainable interventions to address HIV/AIDS in sub-Saharan Africa— and took place in HIV clinics affiliated to the University of Cape Town. The sponsors had no involvement in the study design, analysis, or decision to publish. The study was based on a prospective cohort study—the Cape Town AIDS Cohort (CTAC).

The researchers compared the cost of services for 292 patients who were given HAART with the costs for a matched comparison group (with the same number of patients) who were not given any antiretroviral drugs. Twenty-seven patients in each group had AIDS; the others were HIV-infected but did not have AIDS. The researchers calculated costs per patient year (PPY) and per life-year gained (LYG)—i.e., the total cost divided by the number of extra years the treated patients lived. Calculations were done separately for patients with AIDS and for those without AIDS.

Patients on HAART required fewer hospital admissions. Depending on how long the patient survived and the price of antiretrovirals used, HAART reduced treatment costs for those patients with AIDS. For this group, the cost savings ranged from US$219–US$2,116. For patients without AIDS, the yearly cost of treatment (ranging from US$597–US$1,772) was, in the opinion of the authors, and after taking into account the South African standard of cost of living, considered to be affordable. However, it is expected that South Africa will soon be able to manufacture antiretroviral drugs locally and more cheaply. This would increase the amount saved by introducing HAART.

The study had a number of limitations. Because HAART was not used in routine clinical practice, the researchers had to compare a group of patients enrolled in clinical trials with a control group that was not part of the trials. The study was also confined to the use and cost of services; but when a person is infected with HIV and becomes ill or dies from AIDS, it is clearly not only the health services that face costs. The patient, their family, and the country suffer financially. HAART, as a more effective treatment might also lower these “indirect” costs, but this was not an issue examined here. It is to be hoped that further research includes an evaluation of the indirect costs and benefi ts. Nevertheless, the present study should encourage policymakers in low- and middle-income countries to consider introducing HAART into public-sector health care; reductions in the use of hospital services by patients with HIV could free scarce resources, to the benefi t of all who use the health services.

